# Sexual conflict over remating interval is modulated by the *sex peptide* pathway

**DOI:** 10.1098/rspb.2016.2394

**Published:** 2017-03-01

**Authors:** Damian T. Smith, Naomi V. E. Clarke, James M. Boone, Claudia Fricke, Tracey Chapman

**Affiliations:** 1 School of Biological Sciences, University of East Anglia, Norwich Research Park, Norwich NR4 7TJ, UK; 2 Institute for Evolution and Biodiversity, University of Muenster, Huefferstr. 1, 48149 Muenster, Germany

**Keywords:** sexual conflict, sexual selection, *Drosophila melanogaster*, sperm competition

## Abstract

Sexual conflict, in which the evolutionary interests of males and females diverge, shapes the evolution of reproductive systems across diverse taxa. Here, we used the fruit fly to study sexual conflict in natural, three-way interactions comprising a female, her current and previous mates. We manipulated the potential for sexual conflict by using *sex peptide receptor* (*SPR*) null females and by varying remating from 3 to 48 h, a period during which natural rematings frequently occur. *SPR*-lacking females do not respond to sex peptide (SP) transferred during mating and maintain virgin levels of high receptivity and low fecundity. In the absence of SPR, there was a convergence of fitness interests, with all individuals gaining highest productivity at 5 h remating. This suggests that the expression of sexual conflict was reduced. We observed an unexpected second male-specific advantage to early remating, resulting from an increase in the efficiency of second male sperm use. This early window of opportunity for exploitation by second males depended on the presence of *SPR*. The results suggest that the SP pathway can modulate the expression of sexual conflict in this system, and show how variation in the selective forces that shape conflict and cooperation can be maintained.

## 1. Introduction

Sexual conflict, in which the evolutionary interests of males and females diverge, is a pervasive selective force for driving evolutionary change. It has the capacity to result in evolutionary novelty, population divergence, and reproductive isolation [1]. The rich descriptions of sexual conflict across different taxa, mediated by diverse mechanisms, confirm its general importance [2].

Theory shows that the fitness outcomes among mating partners are likely to depend on the investment made by all interacting individuals, which will be influenced by their ‘knowledge’ and control of each other's investment patterns and the extent to which each individual can exert power [3]. Outcomes can span the whole range of cooperation through to conflict [4]. Intriguingly, there may also be considerable potential for males to hijack the investment of other males [5,6]. Overall, these mating interactions can act as a potent fuel for driving evolutionary change and maintaining genetic variation [7].

The mechanisms by which males and females can enforce their interests include those that influence all aspects of mating investment, including sperm competition. Sperm competition between different males mating with the same female can be a particularly rich source of selection for adaptations that favour success in male–male competition or that reduce its occurrence. For example, there are physical barriers such as the detachable penis in some spiders [8], physical mating plugs in bumblebees and butterflies [9,10], chemical repellents in butterflies [11], and receptivity-inhibiting seminal fluid proteins (Sfps) [12,13]. Adaptations that favour the success of males in sperm competition include those that allow males to increase their numerical superiority of sperm or Sfps inside females according to the threat of sperm competition [3,6,14] and those that allow efficient storage, retention, and high efficiency of sperm use [15].

Sfps have been well studied in the fruit fly *Drosophila melanogaster*. They affect a diverse range of post-mating responses in females, such as egg laying, feeding, immune gene expression, receptivity, siesta sleep, sperm storage, retention, and usage [16–22]. Hence, through manipulating the responses to Sfps we can potentially vary the key factors of investment (e.g. egg laying) and control (e.g. sexual receptivity). Sex peptide (SP) is a Sfp transferred to females during mating that is key in eliciting female post-mating responses of elevated fecundity and decreased female receptivity [19,20,23–25]. It does so by binding to the sex peptide receptor (SPR), which is expressed in various sites in the nervous system and in the female genital tract [23,26–28]. As SP can alter reproductive investment and control, hence potentially the balance of power in mating interactions, it has the potential to mediate sexual conflict. Consistent with this, induction of SP responses can benefit males but exert costs in females [29,30]. Interestingly, consistent with the hypothesis that sexual conflict is likely to generate or maintain genetic variation, different alleles of SPR respond differently to different alleles of SP [31]. This suggests that SP and SPR could be subject to negative frequency dependent selection due to sexual conflict over remating rate.

SPR also influences sperm competition dynamics. First, it delays sperm competition due to its effect on delayed remating in females [19,20]. Second, it influences the release of sperm from storage [15] and hence determines fertilization efficiency and sperm dynamics in the female sperm storage organs. Although a role in the outcome of sperm competition is suggested by these studies, the importance of SPR when a female mates with more than one male is not yet known.

Here, we used the fruit fly model system to measure indices of fitness for each individual when a female mated with two males in series. This design was used to capture some of the natural complexity of sexual competitions that occur simultaneously between multiple individuals and to allow us to simultaneously measure the fitness interests of all interacting parties. We tested the effect of remating interval and the presence or absence of the *sex peptide* pathway. We manipulated remating interval because it is predicted to be a frequent target of sexual conflict, as males benefit from mating with a female immediately, whereas females may pay costs from mating too often [2]. The remating interval was varied from 3 to 48 h and disruption of the *sex peptide* pathway was achieved by using females containing a genetic deletion of the *SPR* locus [23]. To uncover the underlying interactions between the sperm of the different males during these competitions we used red- and green-spermed males [32].

The main result was that manipulating control and investment patterns via removal of the ability to respond to SP led to a convergence of fitness interests, with all parties in the SPR-lacking treatment achieving highest mean progeny production when the remating interval was 5 h. There was also an unexpected second male advantage through the acquisition of extra offspring from early rematings. This was achieved by an increase in the efficiency with which second male sperm were used and was dependent on the presence of *SPR*. This effect reduced the reproductive success of the first male and represents a potential example of exploitation. Taken together, the results show that the *sex peptide* pathway can modulate the extent of sexual conflict and that variation in traits influenced by sexual conflict can be maintained by the shifting reproductive interests of the individuals involved.

## 2. Material and methods

### (a) Fly rearing and stocks

We maintained flies on sugar-yeast-agar (SYA) food at 25°C with a 12 : 12 h light : dark cycle. We collected eggs on agar-grape juice plates, as in [33], and 24 h later, when the eggs hatched, placed 100 larvae into vials (75 mm high and 24 mm diameter) each containing 7 ml SYA medium. This was done to standardize the density of cultures to minimize difference in size and hence individual quality. We collected adult females from these cultures within 6 h of eclosion to ensure they were virgin and collected males within 2 days of eclosion. We housed all flies in single sex groups of 10 for 3–5 days post eclosion before conducting the experiments.

We used *SPR*^0^ females containing the deletion *Df(1)Exel6234* (Bloomington Drosophila Stock Centre #7708), which covers the entire *SPR* locus on the X chromosome (for details see [23]). *SPR*^0^ females from this line do not produce SPR [23]. Prior to use, we backcrossed this stock six times into a *Dahomey[white]* genetic background (Dahomey in which the *w^1118^* mutation had been backcrossed multiple times) to increase vigour. Hence, the *Dahomey[white]* stock was used to generate control, *SPR^+^* females. First and second-mating males were obtained from stocks in which protamine B (a sperm nuclear protein involved in the dense packaging of DNA in sperm) had been tagged with green fluorescent protein (GFP) or dsRed, respectively (as described in more detail in [32]).

### (b) Mating frequency in wild-type flies

We measured natural remating frequencies in two ways. First, we raised flies under standardized density conditions as described above and mated 100 virgin Dahomey females each to a wild-type Dahomey male. After mating, the male was aspirated out and replaced with another wild-type Dahomey male. We observed all females for 8 h and recorded how long it took females to mate for a second time. In the second approach, we raised flies and conducted initial matings as above. After the completion of the first matings, we aspirated out each male and randomly assigned the females to remating treatments in which we challenged females with males 2, 4, 6, 8, 24, and 48 h later. For each remating test, we observed pairs for 30 min and recorded the number that remated. We repeated this assay twice using 22 and 30 females per block, respectively.

### (c) Fitness estimates of rematings in *SPR*^+^ and *SPR*^0^ backgrounds

We used the number of offspring fathered by each male and the total number of offspring produced for females as index of fitness. In this assay, the first male's fitness was determined by the number of offspring he fathered before the female remated and any offspring gained in competition with the second male in the 48 h period after remating. The second male's fitness was the number of offspring fathered in the 48 h period after remating. The estimate of female reproductive output was the total number of offspring produced by the female from the first mating and up to 48 h following remating.

The day before experiments we placed individual females into vials with SYA food. On the morning of the experiment we mated *SPR*^0^ or *SPR^+^* females to a GFP-sperm male. We then assigned females randomly to remate 3 h, 5 h, 24 h, and 48 h later to a dsRed-sperm male. We used these remating timepoints because SP is thought to act within 2 h of mating and for at least 48 h afterwards and because natural rematings occur at high frequency over these time intervals [19,20,24,34]. Remating intervals represented a 2 h second-mating opportunity for the female starting at the times indicated after the first mating (i.e. actual remating intervals of 3–5 h, 5–7 h, 24–26 h, and 48–50 h).

We discarded any pairs that did not mate within 2 h of exposure to each other. GFP and dsRed-sperm males are reported to perform equally well as first male competitors, although dsRed-spermed males do less well as second-mating males [32]. In our experiments, we did not compare GFP and dsRed-sperm males within first or second matings; hence, our interpretation is not affected by this variation. We kept females in SYA food vials at all times and we moved the female to a new vial immediately after and 24 h after the second mating. We assigned paternity to the offspring using GFP expression in the offspring of the GFP- (but not dsRed) tagged males. Initial set-up and final sample sizes are shown in electronic supplementary material, table S1. For the calculations of rate of offspring production, we used number of offspring produced per hour.

### (d) Sperm distribution following rematings in *SPR*^+^ and *SPR*^0^ backgrounds

To determine whether SPR influenced sperm dynamics, we followed the same experimental procedure as the offspring production assay described above, up until the point of the second mating. We then flash froze the females 1 h after the start of remating. This interval is reported to represent the time of maximum sperm storage following mating, or a plateau in sperm storage [32,35]. We then kept females at −80°C until dissection of their reproductive tracts in PBS and collection of the GFP and dsRed-sperm images. We collected images using the Zeiss AxioPlan 2ie microscope using a 20× PlanApochromat objective (0.6 NA) and the Zeiss AxioCam HRc CCD camera. We stitched together multiple images for each reproductive tract using the MosaiX module of the AxioVision software. We excited GFP fluorescence at 470–500 nm and for dsRed at 540–585 nm, and measured emission at 500–560 and 600–650 nm, respectively. We collected a GFP, dsRed, and a bright-field channel for each image and used a macro to split the colour channels and measure the number of fluorescent pixels in each image. We imaged the whole female reproductive tract and designated sperm to the three main sperm-containing components: the bursa, the seminal receptacle, and the spermathecae (sample sizes in electronic supplementary material, table S2).

### (e) Statistical analysis

We used generalized linear models (GLMs) with remating interval, female SPR status, and their interaction as fixed explanatory variables for all analyses. For the paternity data, we used a binomial response variable comprising first male offspring and second male offspring and used binomial (or quasi-binomial if the model was overdispersed) error structure. For the sperm data, we fitted models using the Poisson or quasi-Poisson error structure. To investigate whether the effect of female SPR status differed among female sperm storage organs we used remating interval, female SPR status and sperm storage organ, and all their interactions as fixed effects in a quasi-Poisson GLM, with sperm stored for each individual (first male, second male, and female) as the response variable in separate models. For the proportion data, we calculated the standard error using 
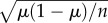
, where *μ* is the sample mean and *n* the sample size. All factors in the statistical models were retained to show the effects of the main factors, which were all experimentally manipulated. We conducted post hoc analysis of offspring production to compare the maximum mean for each female genotype (*SPR^+^* and *SPR*^0^) using the glht function in the multcomp package (v. 1.4-3; [36] in R v. 3.1.3 [37]).

## 3. Results

### (a) Mating frequency

We first examined the natural remating frequency of unmanipulated wild-type females over the remating intervals employed in the main fitness experiments, using two experimental designs. This showed that, when housed continuously with wild-type males, 60% of wild-type females remated within 6 h (electronic supplementary material, figure S1). When females were separated from males for the period between matings and then exposed to males at different time intervals, approximately 30% of females remated after 6 h (electronic supplementary material, figure S2). During the main experiments with SPR control and null females, and red- and green-spermed males, the remating rate ranged from 11 to 88% (electronic supplementary material, figures S3 and S4). Hence, remating was naturally frequent over the time points examined in this experiment. This was supported by additional data on the unmanipulated frequency of rematings of once-mated *SPR^+^* and *SPR*^0^ females with wild-type males (electronic supplementary material, figure S5).

### (b) Fitness estimates

The main experiment comprised *SPR*^0^ or *SPR^+^* females mated first to GFP-sperm males and then remated 3 h, 5 h, 24 h, and 48 h later to dsRed-sperm males. We counted all offspring produced before and up to 48 h after rematings and determined the paternity of offspring after remating by screening progeny for GFP fluorescence. In the second replicate experiment, we flash froze females 60 min after rematings to determine sperm storage patterns.

#### (i) First male fitness

**Figure 1.**
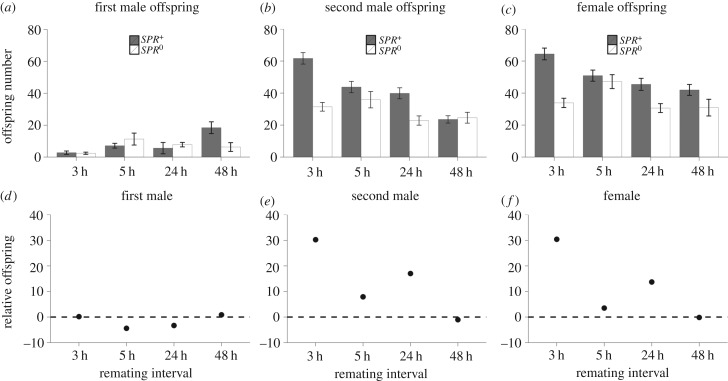
Top row shows the offspring (mean ± s.e.) produced by the (*a*) first and (*b*) second male and (*c*) the female, for each remating interval, in the *SPR^+^* and *SPR*^0^ female backgrounds. Bottom row shows the relative number of offspring produced for *SPR^+^* and *SPR*^0^ treatments for the (*d*) first males, (*e*) second males, and (*f*) female after the second mating. Values above zero indicate more offspring for *SPR^+^* in comparison with *SPR*^0^. The points indicate the difference in treatment means. At each interval, pairs were given 2 h in which to remate, starting at the times indicated (i.e. 3–5 h, 5–7 h, 24–26 h, and 48–50 h).

The fitness of the first male comprised all the offspring he fathered before and after remating. There was a significant interaction between remating interval and female genotype (*SPR*^0^ or *SPR^+^*) on the absolute number of first male offspring produced (quasi-Poisson GLM: *F*_3,114_ = 3.867, *p* = 0.011; figure 1*a* and electronic supplementary material, table S3). Overall, the range of offspring production over all remating intervals in the *SPR*^0^ treatment was smaller than for *SPR^+^*. The maximum mean offspring production was observed at 5 h and 24 h for the *SPR*^0^ and *SPR^+^* treatments, respectively. Post hoc analysis of these maxima compared against the other *SPR*^0^ or *SPR^+^* time points supported the existence of different maxima for control versus SPR-lacking treatments. There was significantly higher progeny production in *SPR^+^* treatments at the 48 h remating interval than at the 3 h and 5 h times, an effect that was not found in *SPR*^0^*,* in which the maximum occurred earlier (figure 5).

#### (ii) Second male fitness

We measured second male fitness as the number of offspring produced in the 48 h after remating. We again found a significant interaction between remating interval and female SPR status (quasi-Poisson GLM: second male *F*_3,201_ = 4.376, *p* = 0.005; figure 1*b* and electronic supplementary material, table S4). Second males produced significantly more offspring when mated to a *SPR^+^* female. The range of offspring production over all remating intervals in the *SPR*^0^ treatment was again smaller than for *SPR^+^*. The maximum mean offspring production was observed at 3 h and 5 h for *SPR^+^* and *SPR*^0^ treatments, respectively. In *SPR^+^*, there was significantly higher progeny production at 3 h remating than at all other time points, an effect that was not found for *SPR*^0^, in which the maximum occurred later (figure 5).

#### (iii) Female fitness

We measured female fitness as the total number of offspring produced overall before and after remating. The number of offspring produced was influenced by a significant interaction between remating interval and female SPR status (quasi-Poisson GLM: *F*_3,201_ = 5.672, *p* < 0.001; figure 1*c* and electronic supplementary material, table S5). The pattern for females showed a similar pattern to that of the second male (*SPR^+^* females achieved maximum mean offspring production at 3 h and *SPR*^0^ females at 5 h). This was attributable to the second male's offspring making up the majority of the total number of offspring produced after remating. In the *SPR^+^* treatment, there was significantly higher offspring production at 3 h remating than at all other remating intervals, an effect that was not present in the *SPR*^0^ treatment, in which maximum offspring occurred later (figure 5).

We also calculated the difference in number of offspring produced between the *SPR^+^* and *SPR*^0^ females after the second mating by each individual at each remating interval (figure 1*d–f*). The dashed line at zero indicates no difference in offspring production by *SPR^+^* and *SPR*^0^ females, above the line represents more offspring from *SPR^+^* females, below the line more offspring from *SPR*^0^ females. The first male produced approximately the same number of offspring after the second mating regardless of whether he mated to a *SPR^+^* or a *SPR*^0^ female (figure 1*d*). However, the second male produced on average 30.24 more offspring if he mated to an *SPR^+^* female in comparison with an *SPR*^0^ female in the 3 h rematings (figure 1*e*). *SPR^+^* females also produced many more offspring than *SPR*^0^ females when they mated 3 h after their initial mating (figure 1*f*). The similarity between the pattern for the second male (figure 1*e*) and the female (figure 1*f*) indicates that the boost in offspring production by *SPR^+^* females remating after 3 h was almost entirely due to the production of second male offspring.

**Figure 2.**
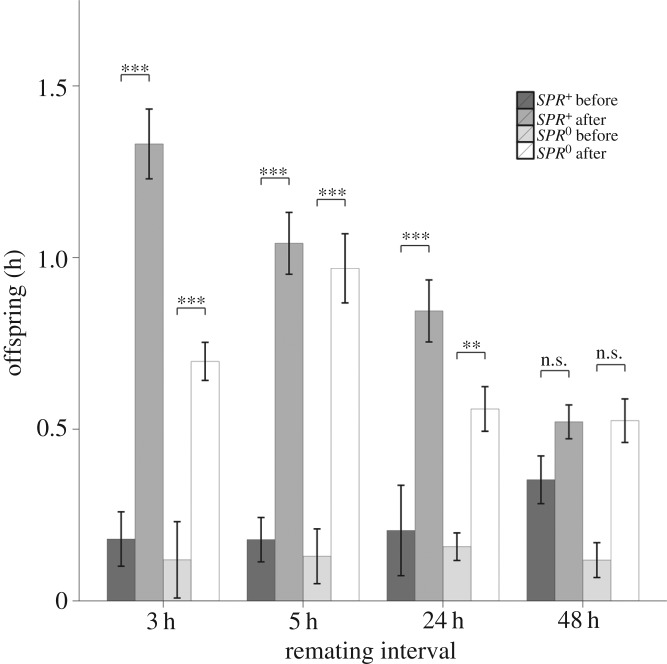
Rate of offspring produced (number of offspring produced per hour) before and after remating for *SPR^+^* and *SPR*^0^ females. At 3, 5, and 24 h remating intervals offspring production was much faster after the second mating than after the first. This was most apparent at 3 h remating in *SPR^+^* females. At each interval, pairs were given 2 h in which to remate, starting at the times indicated (i.e. 3–5 h, 5–7 h, 24–26 h, and 48–50 h).

We were also able to test for differences in the rate (offspring per hour) at which females produced offspring. The rate of offspring production was influenced by a significant interaction between remating interval and before/after remating (Poisson GLM: deviance_3,614_ = 9.293, *p* = 0.026) with females producing offspring at a significantly faster rate after remating in comparison with before (figure 2). Female SPR status also significantly influenced the rate of offspring production (Poisson GLM: *F*_1,625_ = 11.855, *p* < 0.001), with *SPR^+^* females producing offspring at almost twice the rate of the *SPR*^0^ females at the 3 h remating interval (figure 2).

### (c) P2: second male share of paternity

**Figure 3.**
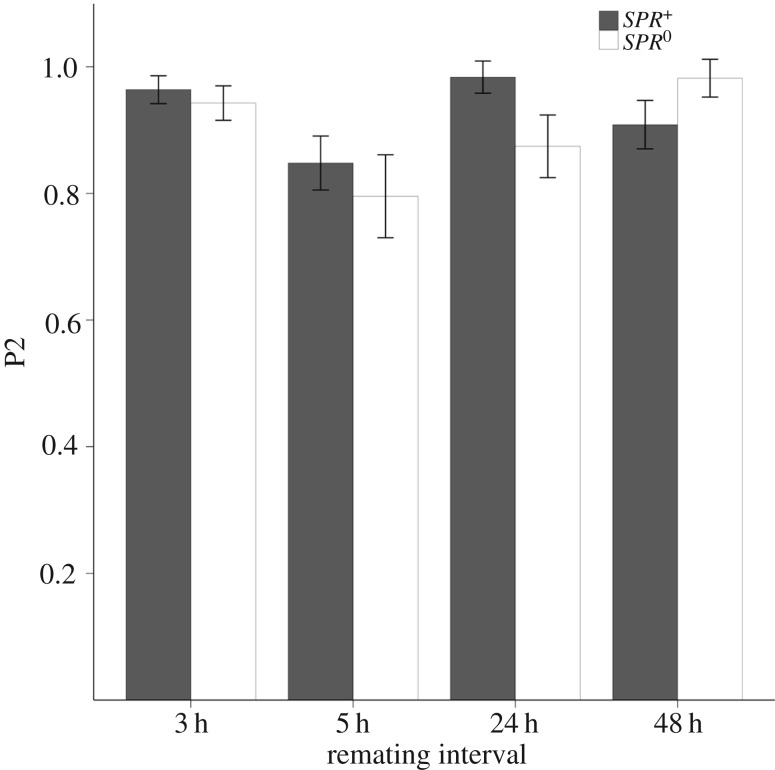
P2 (proportion of second male progeny produced) plotted against remating interval and female SPR status. Both factors were statistically significant predictors of P2. At each interval, pairs were given 2 h in which to remate, starting at the times indicated (i.e. 3–5 h, 5–7 h, 24–26 h, and 48–50 h).

We also examined male fitness as the relative share of offspring produced after remating, or P2 (the proportion of offspring sired by the second male). Both female SPR status (*F*_1,204_ = 7.923, *p* = 0.005) and remating interval (*F*_3,205_ = 8.045, *p* < 0.001) significantly influenced P2, although there was no significant interaction between them (*F*_3,201_ = 1.496, *p* = 0.217). For males mated to *SPR^+^* females, P2 was highest at 24 h remating and for *SPR*^0^ mated males highest at 48 h (figure 3).

### (d) Distribution of sperm in storage

To discover whether there was any signature of the differences in sperm use efficiency, we examined the number of each male's sperm stored in each of the female's sperm storage organs (spermathecae and seminal receptacle) and in the bursa, following remating. We found that the number of each male's sperm stored by the female did not map directly on to the number of offspring or the paternity of the males. Although remating interval and female SPR status significantly influenced the number of each male's sperm stored, these numbers did not match the pattern of the fitness indices (electronic supplementary material, figure S6). Summary ANOVA tables for analysis of the influence of female sperm storage organ, remating interval, and female SPR status on sperm numbers for each individual party can be found in electronic supplementary material, tables S6–S8.

**Figure 4.**
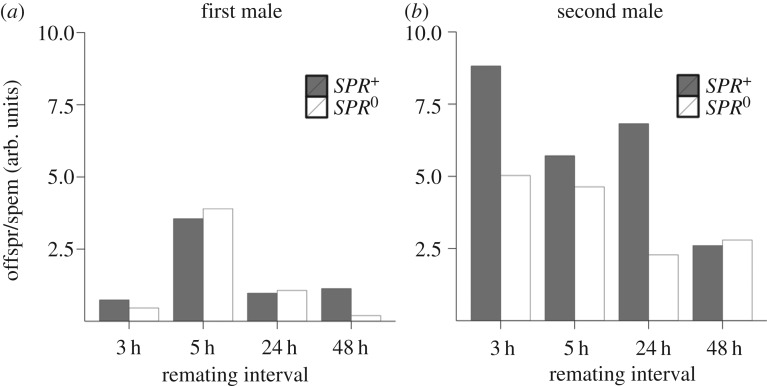
Efficiency of sperm use by the (*a*) first and (*b*) second male, against remating interval and female SPR status. Shown is the number of offspring (‘offspr’) produced per sperm (in arbitrary (‘arb’) units of fluorescence). The second male's sperm was used at a higher efficiency in *SPR^+^* than in *SPR*^0^ females. At each interval, pairs were given 2 h in which to remate, starting at the times indicated (i.e. 3–5 h, 5–7 h, 24–26 h, and 48–50 h).

An analysis of the efficiency of sperm use (number of offspring per unit sperm) indicated that the second male benefitted significantly from early rematings in *SPR^+^* but not *SPR*^0^ females (figure 4*a,b*). At 3 h remating, second males mated to *SPR^+^* females had almost double the number of offspring per unit of sperm stored within females than did second males mated to *SPR*^0^ females. This was even more exaggerated at the 24 h remating interval, but not for the 5 h and 48 h remating treatments (figure 4*b*). First male sperm use efficiency was much less influenced by female SPR status (figure 4*a*).

## 4. Discussion

**Figure 5.**
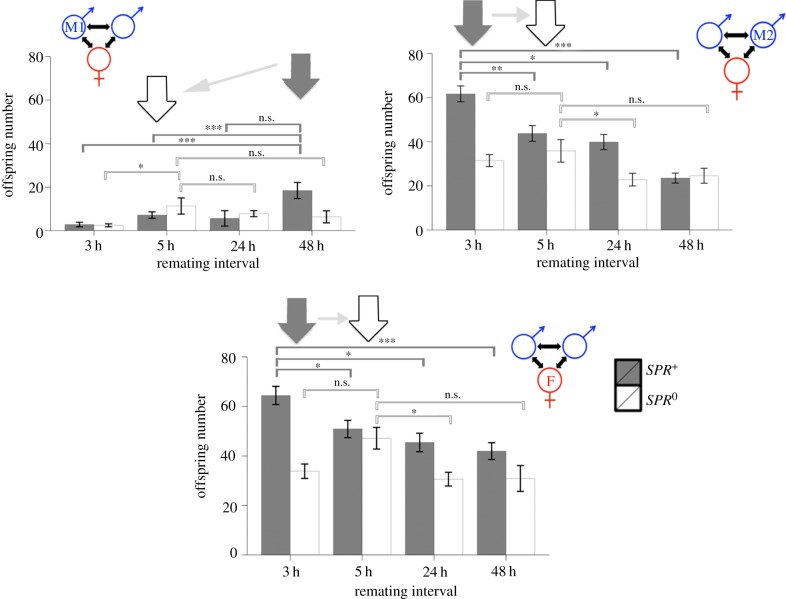
Summary scheme for the reduction in sexual conflict by removal of the *sex peptide* pathway. In controls (grey bars) the fitness optimum for 2nd males (M2) and females (F) differed from that of the 1st male (M1). Maximum mean offspring, for *SPR^+^*, is indicated by the large grey arrows for the first and second males and the female. When we removed the *sex peptide* pathway (using *SPR*^0^ females) the fitness optimum of all individuals converged on the 5 h remating interval. Maximum mean offspring, for *SPR*^0^*,* is indicated by the white arrows, for the first and second males and the female. Hence, sexual conflict was reduced in the absence of *SPR*. At each interval, pairs were given 2 h in which to remate, starting at the times indicated (i.e. 3–5 h, 5–7 h, 24–26 h, and 48–50 h). Post hoc analysis results are indicated by the horizontal brackets comparing the maximum mean offspring production value to all other remating internals within *SPR^+^* (dark grey) and *SPR*^0^ (light grey) treatments. n.s.: not significant, *p < 0.05, **p < 0.01, ***p < 0.001. (Online version in colour.)

Our study shows that the *sex peptide* pathway can determine the balance of fitness interests in mating males and females. By measuring fitness indices for all individuals involved, we showed that when a wild-type female mates with more than one male there was potential conflict over remating intervals. However, in SPR-null females, in which reproductive investment and control were manipulated, this sexual conflict was reduced and the fitness interests of all parties converged (figure 5). The range in mean offspring production was larger for *SPR*^+^ than *SPR*^0^ females across the remating intervals, suggesting that offspring numbers became more similar in the absence of SPR. Furthermore, in the *SPR^+^* background maximum offspring production for the first and second males and females was observed at different time intervals (24/48 h, 3 h, and 3 h, respectively) and became significantly different, and more convergent (at 5 h), for all parties in the *SPR*^0^ background. The results suggest a significant reduction in sexual conflict over remating interval in the absence of SPR.

The remating peak of *SPR*^0^ females (steepest rise in the cumulative remating curve) occurred at approximately 100–120 min (approx. 2 h) and for *SPR^+^* females later, at approximately 220–250 min (approx. 4 h) (electronic supplementary material, figure S5). In the wild-type condition, the first male gained more paternity from longer remating intervals during the 3–5 h timepoints, and the female plus the second male from earliest rematings (figure 5). Hence, natural remating intervals in the *SPR^+^* condition might seem to favour the interests of the first male, with the opposite being true in *SPR*^0^ females. However, in this study we considered the fitness of all parties following rematings, i.e. the situation in which both males were engaged in sperm competition. We did not include additional fitness gained by the first male (and potentially lost by the second) due to the prevention of remating. This would be very interesting to investigate further and would also aid in the development of formal theory.

Pre-requisities for sexual conflict over remating in this system were evident, as variation in remating interval led to different fitness outcomes (figure 1). In addition, sexual conflict for the different individuals was evident because in the control conditions the first male had a different optimum remating interval than the female or the second male. However, when we prevented females from responding to SP, this sexual conflict was reduced as all individuals maximized their fitness at a similar (approx. 5 h) remating interval.

SP is known to reduce female remating rate and increase egg production [19,20,23–25] and plays a role in sexual conflict over the frequency of remating [29,30]. In this study, we showed that the *sex peptide* pathway can determine the extent of sexual conflict over optimum remating interval for individuals involved in mating interactions under polyandry. These data support the hypothesis that the *sex peptide* pathway contributes to the expression of sexual conflict [29,30].

An additional finding was the role of SPR in determining the number of offspring fathered by the second male in early rematings. At the 3 h remating interval, males fathered significantly more offspring following rematings to *SPR^+^* in comparison with *SPR*^0^ females. This effect was not observed at any other remating interval (figure 1). This finding could have two explanations: (i) the second male could hijack the SP transferred by the first male. Having replaced the first male's sperm with his own, the second male might exploit the effects of first male transferred-SP to boost his fertilization efficiency. Second-mating males might also hijack the effects of other Sfps [5], as the rate of offspring production was higher in *SPR*^0^ females after remating, in comparison with before remating. Consistent with this, Chapman *et al.* [38] previously showed that transfer of another Sfp, Acp36DE, by the first male can be exploited by later mating males to boost their sperm storage and fertilization success. Future tests of these ideas would be useful. (ii) The second male could also be responding to sperm competition by transferring more SP to a mated than virgin female. However, although males can respond to female mating status by adjusting their sperm and other Sfp allocation for at least 4 days after the female's previous mating [6,14], they appear not to adjust SP allocation if a female mated 24 h previously [6]. Therefore, males can strategically adjust their ejaculate over the time periods we studied, but they appear not to adjust SP allocation. The evidence suggests it is unlikely that the second male transferred more SP in rematings occurring at 3 h.

Early remating appears to be frequent in this species [39–42] (electronic supplementary material, figures S1–S5). The magnitude of this early remating is likely to have been unappreciated to date because longer remating intervals are typically used in sperm competition assays [19,43–45]. The high frequency of early rematings that we and others have observed suggest that this is a real phenomenon in the laboratory and field, facilitated by high densities and continuous exposure to the opposite sex either in population cages or in patchy high density field aggregations. In these experiments, we used a design of random cohorts remating at discrete time points to ensure experimental control and avoid the bias of variation in female remating propensity. However, in the future it would also be interesting to study further the outcomes of unmanipulated natural early rematings.

Variation in male and female quality was minimized by the use of standardized density culturing in the experiments. Hence, though we cannot rule out that variation in the proportion of remating relates to differences in individual quality, with the potential for associated bias, these effects are expected to be minimal. In addition, there was no simple correlation between proportion remating and fitness in sperm competition. For example, the proportion of individuals remating with *SPR*^0^ females was similar 3–5 h (0.25, 0.22) yet there was a fitness difference arising from differing sperm competition outcomes across those intervals (e.g. for male 1; figure 5). A second example is that the proportion of rematings with *SPR^+^* females differed over 24–48 h (0.41–0.73), yet the sperm competition outcomes were similar (figure 5).

Our data support the idea that rather than representing a ‘switch’ mechanism [23,28,46–49] female responses to SP show dose dependency. We observed that two matings in close succession caused an additional increase in the SP response. However, if those doses of SP were received a little further apart, there was no additional fecundity boost, consistent with previous observations [6]. We suggest that females may exhibit time-dependent insensitivity to the receipt of additional SP. This could benefit females in order to avoid over investment in current offspring at the expense of future reproductive efforts.

The distribution of stored sperm did not map on to the pattern of offspring production observed. Generally, the number of second male sperm stored was higher than for first males. This was expected because of the widely observed last male precedence in this species. It is clear that differences in the efficiency of sperm use for fertilization occurred and did not leave a signature in the pattern of stored sperm. When males fathered significantly more offspring in rematings with *SPR^+^* females at 3 h, it was the number of offspring produced per unit of stored sperm that appeared to differ, rather than the number of stored sperm number overall. The SP pathway is known to play a role in release of sperm from the storage organs during fertilization [15], which we predict should influence the efficiency of sperm use (as is true for other Sfps [50]). Whether the efficiency of sperm usage is subject to sexual conflict is not yet known, though this trait is predicted to influence the evolution of the *sex peptide* pathway.

Our study fits into a wider context of the observed importance of sexual conflict between the interests of males and females across a wide range of taxa [2]. It broadens our knowledge to encompass measurement of the fitness interests of multiple parties over varied time scales. In this, it represents the natural complexity of polyandrous mating systems and recognizes the importance of the timing of rematings. The reduction of conflict through removal of SPR, which acts via the alteration of investment (egg production) and control (sexual receptivity), is important as it shows that whether there is conflict or cooperation depends upon the balance of interests between all the parties involved. Hence, conflict is not a fixed property within species. It predicts that other ecological factors that can alter the balance of investment patterns should also influence the expression of conflict. Such an effect is indeed observed upon manipulation of the nutritional environment [30]. We show that males can also increase their fitness not only by being the first and only male to mate, but by mating second and exploiting investment from previous males.

The evolutionary conflict between males and females is an inevitable consequence of the same genome serving the fitness optima of different sexes. Here, we describe how the timings of matings and the molecular pathways involved can influence the conflict between the sexes, and in turn, influence the evolutionary dynamics of sexual organisms. The evolution of the *sex peptide* pathway has been shaped by male manipulation of female reproductive output. By severing this pathway, we have shown that it is possible to reduce the sexual conflict between competing males for access to fertilizations.

## Supplementary Material

Combined ESM Figures S1-S6, Tables S1-S8

## Data accessibility

Data available from the Dryad Digital Repository, doi:10.5061/dryad.h5346 [51].
