# Supplementary material for: Sexual conflict over remating interval is modulated by the sex peptide pathway
Source: Proc Biol Sci. 2017 Mar 1;284(1850):20162394. doi: 10.1098/rspb.2016.2394 (PMC5360916; doi:10.1098/rspb.2016.2394)
Supplement: Combined ESM Figures S1-S6, Tables S1-S8 [file rspb20162394supp1.pdf]

# Sexual conflict over remating interval is modulated by the sex peptide pathway

Damian T. Smith[1], Naomi V. E. Clarke[1], James M. Boone[1], Claudia Fricke[2], Tracey Chapman[1]*

[1] School of Biological Sciences, University of East Anglia, Norwich Research Park, Norwich, NR4 7TJ, UK

[2] Institute for Evolution and Biodiversity, University of Muenster, Huefferstr. 1, 48149 Muenster, Germany

Phone: + 44 (0)1603 593210

FAX: + 44 (0)1603 592250

*Author for correspondence: Tracey Chapman

e-mail: tracey.chapman@uea.ac.uk

# Electronic Supplementary Information

Figures S1-S6.

Tables S1-S8.

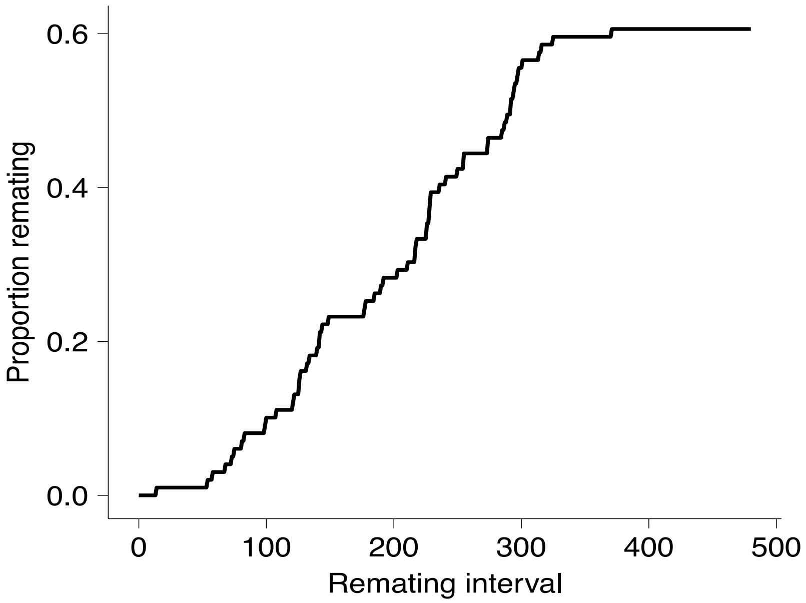


**Figure S1.** Cumulative remating frequency of wild type females under continuous male exposure. Shown is the cumulative proportion of wild type females remating, against time in minutes, when continuously exposed to wild type males following first matings with wild type males. The data show that ~60% of females remated within 6 hours (360 minutes).

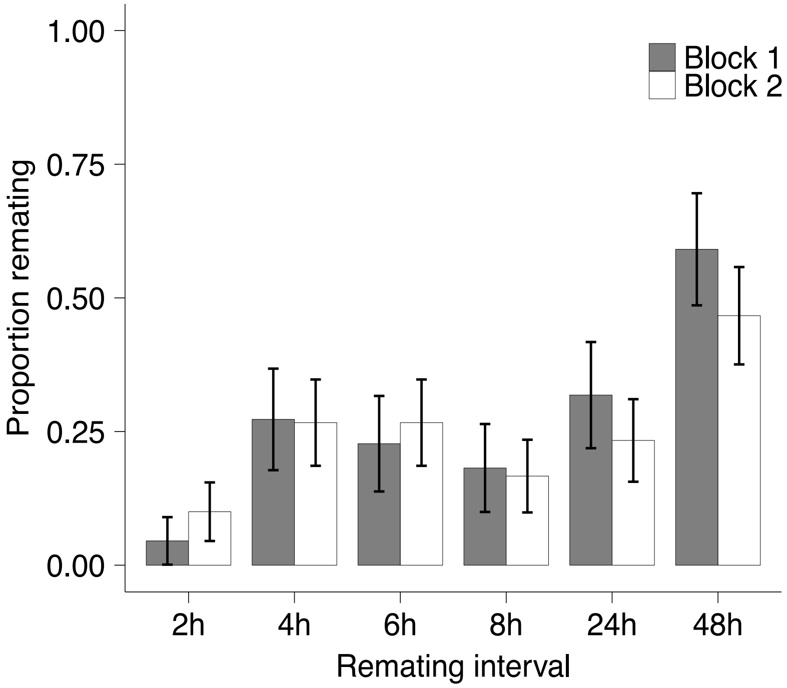


**Figure S2.** Proportion of wild type females remating at different remating intervals in remating tests. Shown are two experimental blocks (block 1,2) of the proportion of wild type females that remated with wild type males at the remating intervals shown, after first mating with wild type males. ~30% of females remated as early as 4h after their first mating.

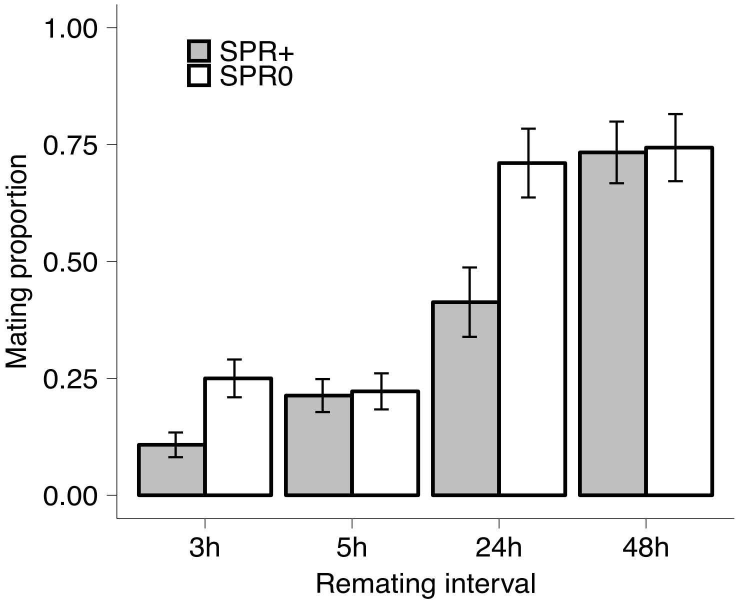


**Figure S3.** Proportion of individuals that remated at different remating intervals in the main fitness experiment**.** The proportion of control ($SPR^{+}$, grey bars) or $SPR^{0}$ (white bars) females in the main fitness experiment that remated with dsRed-sperm males at the remating intervals shown, following first matings with GFP-sperm males. Consistent with the data from the wild type and $SPR^{+/0}$ experiments (figures S1, S2, S4, S5) a high proportion of females remated at the early time points tested, even in the control females.

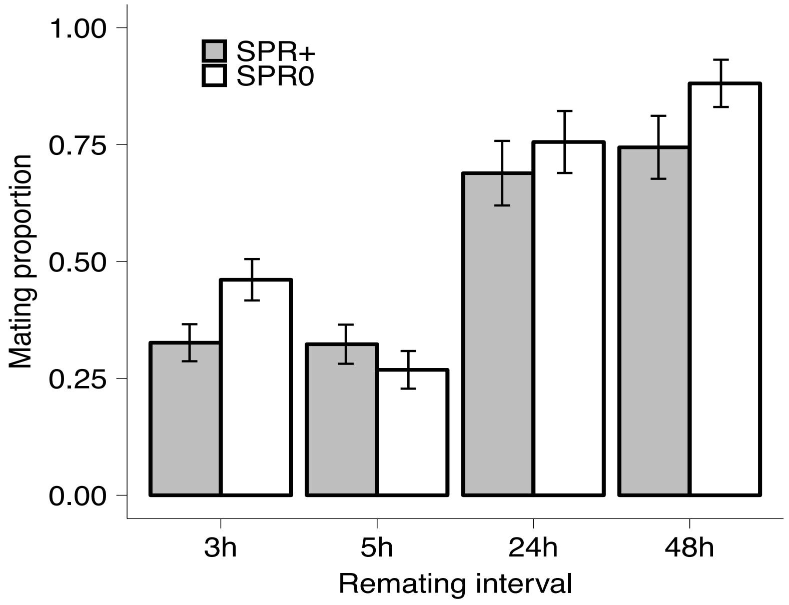


**Figure S4.** Proportion of individuals that remated at different remating intervals for the sperm count experiment. The proportion of control (*SPR*$^{+}$, grey bars) or *SPR*$^{0}$ (white bars) females in the sperm count experiment that remated with dsRed-sperm males at the remating intervals shown, following first matings with GFP-sperm males. Consistent with the data from the wild type and *SPR*$^{+/0}$ experiments (Figures S1, S2, S3, S5) a high proportion of females remated at the early time points tested, even in the control females.

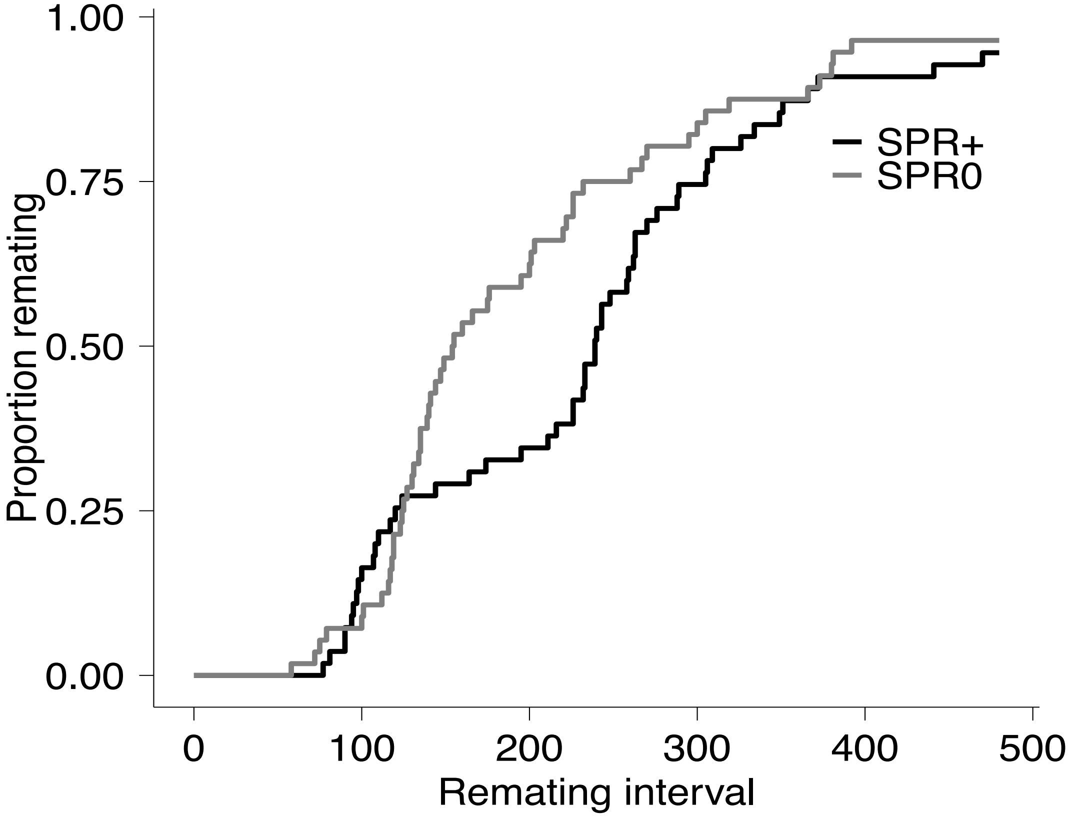


**Figure S5**. Cumulative remating frequency of unmanipulated ("natural") rematings by once-mated *SPR*$^{+}$ and *SPR*$^{0}$ females continuously exposed to wild type males.

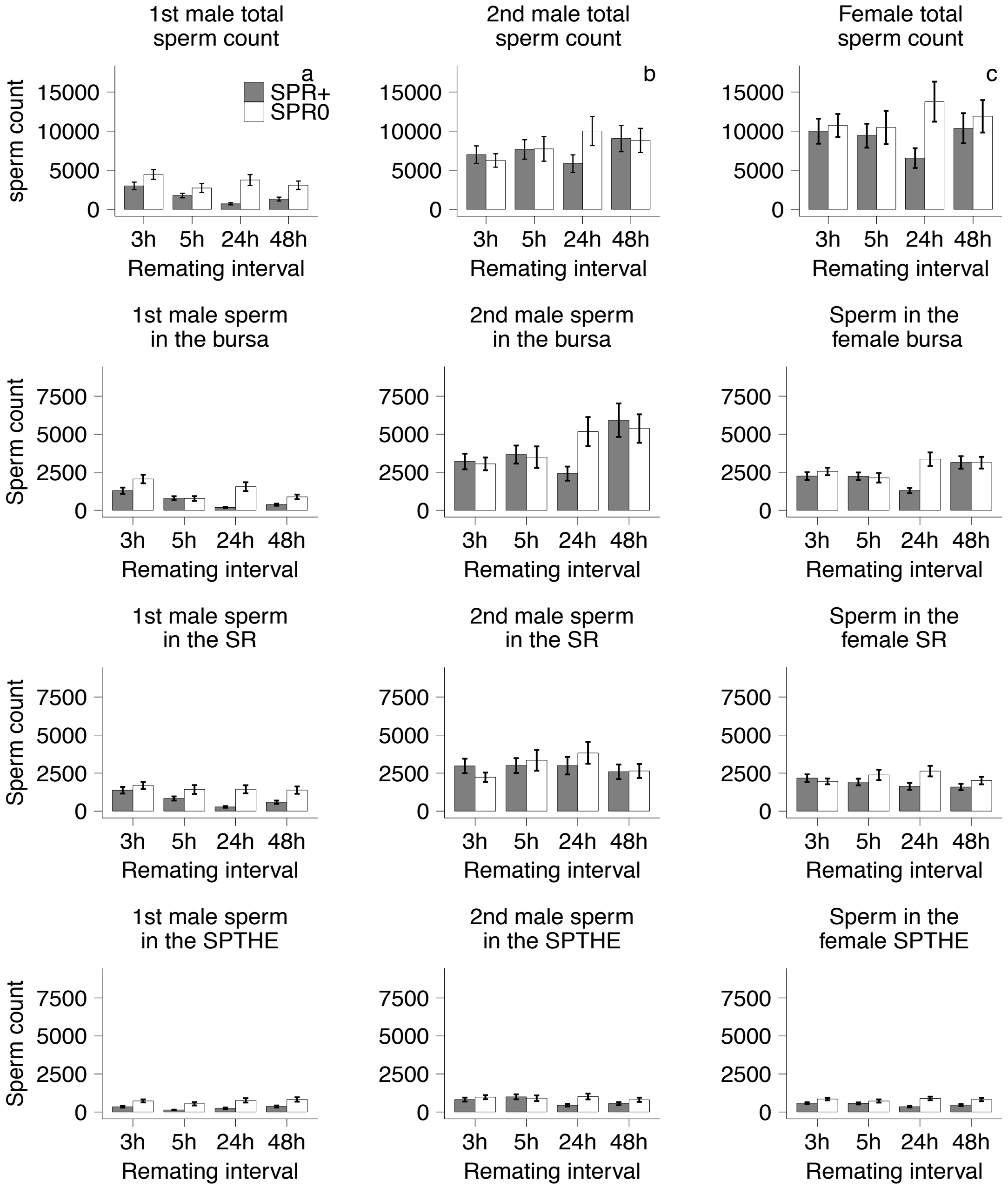


**Figure S6. Number of sperm from the first and second male in storage in the female.** The number of sperm is in arbitrary units of fluorescence derived from the number of fluorescent pixels in each of the captured images (mean ±se). Shown in the LH column in each case is the sperm count for the first male (green fluorescence), in the middle column the count for the second male (red fluorescence) and in the RH column the total sperm count (total fluorescence). The top row shows the sperm counts for all the sperm

storage organs plus the bursa, the second row the sperm counts for the bursa only, the third for the seminal receptacle (SR) only and the bottom row for the two spermathecae combined (SPTHE). The results show that there was little correlation between the pattern of sperm storage and offspring paternity / numbers shown in figure 1 in the main text.

**Table S1.** The number of flies that were initially setup, that mated, that did not mate (unmated), that were lost during the setup and the mating success, for each remating timepoint ('time') and female *SPR* status in the main fitness experiment.

| time | $SPR^{+/0}$ | setup | mated | unmated | lost | mating success | se |
|---|---|---|---|---|---|---|---|
| 0h | $SPR^{+}$ | 400 | 336 | 22 | 12 | 0.84 | 0.02 |
| 0h | $SPR^{0}$ | 400 | 310 | 88 | 2 | 0.78 | 0.02 |
| 3h | $SPR^{+}$ | 139 | 15 | 123 | 1 | 0.11 | 0.03 |
| 3h | $SPR^{0}$ | 116 | 29 | 86 | 1 | 0.25 | 0.04 |
| 5h | $SPR^{+}$ | 136 | 29 | 106 | 1 | 0.21 | 0.04 |
| 5h | $SPR^{0}$ | 117 | 26 | 90 | 1 | 0.22 | 0.04 |
| 24h | $SPR^{+}$ | 46 | 19 | 25 | 2 | 0.41 | 0.07 |
| 24h | $SPR^{0}$ | 38 | 27 | 11 | 0 | 0.71 | 0.07 |
| 48h | $SPR^{+}$ | 45 | 33 | 12 | 0 | 0.73 | 0.07 |
| 48h | $SPR^{0}$ | 39 | 29 | 8 | 2 | 0.74 | 0.07 |

**Table S2.** The number of flies that were initially setup, that mated, that did not mate (unmated), that were lost during the setup and the mating success, for each remating timepoint ('time') and female *SPR* status in the main sperm storage experiment.

| time | $SPR^{+/0}$ | setup | mated | unmated | lost | mating success | se |
|---|---|---|---|---|---|---|---|
| 0h | $SPR^{+}$ | 400 | 357 | 22 | 21 | 0.89 | 0.02 |
| 0h | $SPR^{0}$ | 400 | 338 | 37 | 25 | 0.84 | 0.02 |
| 3h | $SPR^{+}$ | 141 | 46 | 94 | 1 | 0.33 | 0.04 |
| 3h | $SPR^{0}$ | 128 | 59 | 68 | 1 | 0.46 | 0.04 |
| 5h | $SPR^{+}$ | 130 | 42 | 83 | 5 | 0.32 | 0.04 |
| 5h | $SPR^{0}$ | 123 | 33 | 88 | 2 | 0.27 | 0.04 |
| 24h | $SPR^{+}$ | 45 | 31 | 14 | 0 | 0.69 | 0.07 |
| 24h | $SPR^{0}$ | 45 | 34 | 8 | 3 | 0.76 | 0.07 |
| 48h | $SPR^{+}$ | 43 | 32 | 10 | 1 | 0.74 | 0.07 |
| 48h | $SPR^{0}$ | 42 | 37 | 4 | 1 | 0.88 | 0.05 |

**Table S3**. ANOVA table for first male offspring production with remating interval (‘time’) and female *SPR* status (‘SPR’) and their interactions as explanatory factors.

| | Df | Deviance | Resid. Df | Resid. Dev | F | Pr(>F) |
|---|---|---|---|---|---|---|
| NULL | NA | NA | 121 | 1484.360 | NA | NA |
| time | 3 | 272.767 | 118 | 1211.593 | 8.885 | 0.000 |
| SPR | 1 | 6.100 | 117 | 1205.493 | 0.596 | 0.442 |
| time:SPR | 3 | 118.700 | 114 | 1086.794 | 3.867 | 0.011 |

**Table S4**. ANOVA table for second male offspring production with remating interval ('time') and female *SPR* status ('SPR') and their interactions as explanatory factors.

| | Df | Deviance | Resid. Df | Resid. Dev | F | Pr(>F) |
|---|---|---|---|---|---|---|
| NULL | NA | NA | 208 | 2807.138 | NA | NA |
| time | 3 | 464.178 | 205 | 2342.960 | 17.746 | 0.000 |
| SPR | 1 | 368.103 | 204 | 1974.857 | 42.219 | 0.000 |
| time:SPR | 3 | 114.450 | 201 | 1860.407 | 4.376 | 0.005 |

**Table S5**. ANOVA table for female offspring production with remating interval ('time') and female *SPR* status ('SPR') and their interactions as explanatory factors.

| | Df | Deviance | Resid. Df | Resid. Dev | F | Pr(>F) |
|---|---|---|---|---|---|---|
| NULL | NA | NA | 208 | 99190.47 | NA | NA |
| time | 3 | 6704.451 | 205 | 92486.02 | 6.258 | 0.000 |
| SPR | 1 | 14634.439 | 204 | 77851.58 | 40.983 | 0.000 |
| time:SPR | 3 | 6076.582 | 201 | 71775.00 | 5.672 | 0.001 |

**Table S6**. ANOVA table showing the analysis of first male sperm numbers (arbitrary units of fluorescence) stored in the female reproductive tract with sperm storage organ (‘tissue’), remating interval (‘time’) and female *SPR* status (‘SPR’) and their interactions as explanatory factors.

| | Df | Deviance | Resid. Df | Resid. Dev | F | Pr(>F) |
|---|---|---|---|---|---|---|
| NULL | NA | NA | 815 | 1830577 | NA | NA |
| time | 3 | 55356.625 | 812 | 1775220 | 6.466 | 0.000 |
| SPR | 1 | 76245.565 | 811 | 1698975 | 26.717 | 0.000 |
| tissue | 2 | 88035.587 | 809 | 1610939 | 15.424 | 0.000 |
| time:SPR | 3 | 28283.131 | 806 | 1582656 | 3.304 | 0.020 |
| time:tissue | 6 | 20957.835 | 800 | 1561698 | 1.224 | 0.292 |
| SPR:tissue | 2 | 1391.093 | 798 | 1560307 | 0.244 | 0.784 |
| time:SPR:tissue | 6 | 10357.879 | 792 | 1549949 | 0.605 | 0.727 |

**Table S7.** ANOVA table for second male sperm numbers (arbitrary units of fluorescence) stored in the female reproductive tract with sperm storage organ ('tissue'), remating interval ('time') and female SPR status ('SPR') and their interactions as explanatory factors.

| | Df | Deviance | Resid. Df | Resid. Dev | F | Pr(>F) |
|---|---|---|---|---|---|---|
| NULL | NA | NA | 815 | 2998814 | NA | NA |
| time | 3 | 28323.921 | 812 | 2970490 | 3.492 | 0.015 |
| SPR | 1 | 2992.911 | 811 | 2967497 | 1.107 | 0.293 |
| tissue | 2 | 613738.427 | 809 | 2353759 | 113.501 | 0.000 |
| time:SPR | 3 | 29962.817 | 806 | 2323796 | 3.694 | 0.012 |
| time:tissue | 6 | 46521.726 | 800 | 2277274 | 2.868 | 0.009 |
| SPR:tissue | 2 | 3964.935 | 798 | 2273309 | 0.733 | 0.481 |
| time:SPR:tissue | 6 | 9772.519 | 792 | 2263537 | 0.602 | 0.729 |

**Table S8**. ANOVA table for total sperm numbers (arbitrary units of fluorescence) stored in the female reproductive tract with sperm storage organ ('tissue'), remating interval ('time') and female SPR status and their interactions as explanatory factors.

| | Df | Deviance | Resid. Df | Resid. Dev | F | Pr(>F) |
|---|---|---|---|---|---|---|
| NULL | NA | NA | 815 | 3280204 | NA | NA |
| time | 3 | 5640.558 | 812 | 3274563 | 0.598 | 0.616 |
| SPR | 1 | 35197.246 | 811 | 3239366 | 11.194 | 0.001 |
| tissue | 2 | 660016.339 | 809 | 2579350 | 104.950 | 0.000 |
| time:SPR | 3 | 43634.068 | 806 | 2535715 | 4.626 | 0.003 |
| time:tissue | 6 | 24901.855 | 800 | 2510814 | 1.320 | 0.246 |
| SPR:tissue | 2 | 7993.723 | 798 | 2502820 | 1.271 | 0.281 |
| time:SPR:tissue | 6 | 13777.527 | 792 | 2489042 | 0.730 | 0.625 |
